# Thyroid and Hepatic Haemodynamic Alterations among Egyptian Children with Liver Cirrhosis

**DOI:** 10.5402/2012/595734

**Published:** 2012-07-30

**Authors:** Zeinab A. El-Kabbany, Rasha T. Hamza, Ahmed S. Abd El Hakim, Lamis M. Tawfik

**Affiliations:** ^1^Department of Pediatrics, Faculty of Medicine, Ain Shams University, Cairo 11371, Egypt; ^2^Department of Radiology, Faculty of Medicine, Ain Shams University, Cairo 11371, Egypt; ^3^Department of Clinical Pathology, Faculty of Medicine, Ain Shams University, Cairo 11371, Egypt

## Abstract

*Background*. Alterations in thyroid hormones regulation and metabolism are frequently observed in patients with cirrhosis. *Aims*. To assess alterations in thyroid volume (TV), haemodynamics, and hormones in patients with cirrhosis and their relation to hepatic arterial haemodynamics, and disease severity. *Methods*. Forty cirrhotic patients were compared to 30 healthy subjects regarding TV, free triiodiothyronine (fT_3_), free tetraiodothyronine (fT_4_), thyroid stimulating hormone (TSH), and pulsatility and resistance indices in the inferior thyroid and hepatic arteries. *Results*. TV (*P* = 0.042), thyroid volume standard deviation score (TVSDS, *P* = 0.001), Inferior Thyroid Artery Pulsatility Index (ITAPI, *P* = 0.001), Inferior Thyroid Artery Resistance Index (ITARI, *P* = 0.041), Hepatic Artery Pulsatility Index (HAPI, *P* = 0.029) and Hepatic Artery Resistance Index (HARI, *P* = 0.035) were higher among cases being highest in Child-C patients. FT_3_ was lower in patients than controls (*P* = 0.001) and correlated negatively with ITAPI (*r* = −0.71, *P* = 0.021) and ITARI (*r* = −0.79, *P* = 0.011). ITAPI and ITARI correlated directly with HAPI and HARI (*r* = 0.62, *P* = 0.03, and *r* = 0.42, *P* = 0.04, resp.). *Conclusions*. Thyroid is involved in the haemodynamic alterations of cirrhosis. Routine study of thyroid by Doppler and assessment of thyroid functions should be performed in patients with cirrhosis to offer proper treatment if needed.

## 1. Introduction

 Liver cirrhosis is an irreversible alteration of the liver architecture which occurs in response to chronic liver injury from a variety of causes including toxins, chronic viral infection, cholestasis, and metabolic disorders. It consists of diffuse fibrosis of hepatic parenchyma resulting in nodule formation [1].

 Cirrhosis is characterized by complex changes in the systemic and splanchnic haemodynamics [2]. Within the splanchnic and systemic circulation, there is increased cardiac output and hyperdynamic circulation that contributes to increased flow into the portal circulation thereby perpetuating portal hypertension [3]. Vasodilatation occurs mainly, but not only, in the splanchnic area causing blood congestion [4]. By contrast, in other areas such as the kidney and the thyroid gland, the resistance to arteriolar flow increases and blood perfusion of the organ decreases progressively [5]. Moreover, cirrhosis causes increased intrahepatic resistance resulting from both vascular factors and fibrosis [3].

 Alterations in thyroid hormone regulation and metabolism are frequently observed in patients with liver cirrhosis with decreased serum T_3_ concentration probably due to impaired conversion of T_4_ to T_3_ in the liver. In severe cases, low T_4_ concentrations are also observed [6].

 Thyroid hormones affect the vascular system, T_3_ decreases systemic vascular resistance by dilating resistance arterioles of the peripheral circulation through a direct effect on vascular smooth muscle cells. Therefore, alterations in T_3_ may participate in the haemodynamic alterations of cirrhosis [7].

 In medical application, ultrasonic Doppler equipment can be employed for detection and evaluation of the characteristics of blood flow in arteries and veins with the ultrasound beam directed toward the vessel of interest. The sources of echo signals are the red blood cells flowing in the vessel [8].

 With this background, we were stimulated to assess thyroid haemodynamic and hormonal alterations in patients with liver cirrhosis and their relation to hepatic arterial haemodynamics and disease severity.

## 2. Subjects and Methods

### 2.1. Study Population

 This cross-sectional case-control study was conducted over a period of 1 year from the beginning of August 2008 to the end of July 2009. It included 40 Egyptian patients with liver cirrhosis, regularly attending the Pediatric Hepatology Clinic, Children's Hospital, Ain Shams University, Cairo, Egypt. They were 22 males and 18 females with a mean age of 7.8 ± 5.5 years (range: 0.5–16 years).

 The diagnosis of cirrhosis was based on liver histology and clinical examination together with presence of portal hypertension (varices, ascites, and encephalopathy). The etiology of cirrhosis was posthepatitic in 5 patients (2 posthepatitis B and 3 posthepatitis C), glycogen storage disease in 8 patients, biliary atresia in 7 patients, Niemann Pick disease in 6 patients, autoimmune hepatitis in 4 patients, Budd Chiari syndrome in 6 patients, and Wilson's disease in 4 patients.

 Patients were classified according to Child-Turcotte- Pugh classification [9] which uses clinical and laboratory information to stratify disease severity, surgical risk, and overall prognosis. Risk (grade) is based on the total number of points scored and is classified into: low (A): 5-6, moderate (B): 7–9, and high (C): 10–15 points. In group A: no encephalopathy or ascites, bilirubin <1.5 mg%, albumin >3.5 g/dL, prothrombin time 10–14 seconds, group B: mild to moderate encephalopathy, slight ascites, bilirubin 1.5–3 mg%, albumin 2.8–3.5 g/dL, prothrombin time 14–16 seconds, group C: encephalopathy, moderate ascites, bilirubin >3 mg%, albumin <2.8 g/dL, prothrombin time >16 seconds.

 Group A included 15 patients (9 males and 6 females) with a mean age of 6.6 ± 4.5 years (range: 1–14 years), group B included 15 patients (8 males and 7 females) with a mean age of 5.9 ± 6.2 years (range: 0.5–15 years), and group C included 10 patients (5 males and 5 females) with a mean age of 7.2 ± 3.0 years (range: 1–16 years).

 Patients were also classified into ascitic (*n* = 12) and nonascitic groups (*n* = 28) based on clinical examination and ultrasound assessment [10].

 Patients were compared to 30 age- sex- and pubertal-stage-matched apparently healthy children (17 males and 13 females) whose mean age was 7.2 ± 2.0 years ranging between 1–15 years.

 Children with primary thyroid disorders or positive family history of thyroid disorders or history of intake of thyroid modifying drugs in the past 3 months or those who had abnormal thyroid functions or living in areas known to be iodine deficient were excluded from the study.

 An informed written consent was signed up by the parents or caregivers of all studied subjects before enrollment in the study. This study was approved by the Bioethical Research Committee, Faculty of Medicine, Ain Shams University hospitals, Cairo, Egypt. 

### 2.2. Study Measurements

All children were subjected to the following

- Full medical history especially age, sex, residence, history of drug intake, and positive family history of thyroid disorders.
- Examination for signs of hypothyroidism and neck examination to detect presence of goiter if present.
- Thyroid function tests, including serum fT_3_, fT_4_, and TSH with the Immulite 2000 Analyzer using chemiluminescent immunometric assay [11].
- Liver function tests: which included Alanine amino transferase (ALT, IU/L), Aspartate amino transferase (AST, IU/L), serum bilirubin (total and direct, mg%), total proteins (gm/dL), and albumin (gm/dL) using Automated assay, Synchron clinical system, Beckman.
- Doppler ultrasound: using a color Doppler of Power Vision SSA-380A system (Toshiba medical system Co, Ltd. Tokyo, Japan) with muti-Hertz (3 to 5 MHz) convex and sector-pulsed probes. This was carried out on:
  1. Thyroid gland: TV in mL was calculated by ellipsoid method (length × width × thickness × 0.523) [12]. The total volume was calculated as the sum of the volumes of the 2 lobes. TV SDS was calculated, and thyroid enlargement was diagnosed when thyroid volume for age was >2 SD above the mean [13]. After a longitudinal scan of the thyroid lobe, the inferior thyroid artery was identified using color Doppler. The sample volume of Doppler system was placed where the artery lies longitudinally behind the posterior surface of thyroid lobe and the blood flow velocity wave form was analyzed using an angle between 30 and 60-degrees.
  2. Liver: Sonographic examination was carried out 8 hours after the last meal. Color Doppler allowed identification of hepatic artery which was evaluated by an intercostal approach via demonstration of right and left portal veins under a 60 degree angle. In some subjects, hepatic artery traces were seen at least 2 cm away from right to left portal vein bifurkation, therefore, measurements were done from there. Doppler system was placed inside this vessel at an angle of less than 60 degree between the vessel and ultrasound beam and the blood flow velocity waveform was recorded.

In both inferior thyroid and hepatic arteries, resistance and pulsatility indices were calculated according to the following formulae [14]:

(1) Resistance  index  (RI)=  maximum  systolic  velocity−end  diastolic  velocityMaximal  velocity,Pulsatility  index  (PI)=maximum  systolic  velocity−minimal  velocityMean  velocity

RI and PI detect the degree of resistance of blood flow through the blood vessel so when RI and PI are high, this denotes increased vascular resistance of the examined vessel, and vice versa [14]. A value of more than 0.58 for resistance index and 1.0 for pulsatility index was considered high [15].

### 2.3. Statistical Methods

 The data were statistically analyzed using SPSS statistical package version 10 (Echosoft corp, USA, 2006). Description of quantitative variables was in the form of mean ± SD, median, and range, while that of qualitative variables was given as frequency and percentage. Student *t*-test of 2 independent samples was used to compare 2 quantitative groups for parametric data while Mann-Whitney test (*z*-test) was used to compare 2 quantitative groups for nonparametric data. Pearson correlation coefficient (*r*-test) was used to relate different variables to each other. A value of *P* < 0.05 was considered significant.

## 3. Results

 None of our cases had signs of hypothyroidism nor visible or palpable goiter on clinical examination. TV and its SDS and thyroid and hepatic haemodynamic parameters were significantly higher, while fT_3_ was significantly lower among patients than controls with a nonsignificant difference regarding fT_4_ and TSH (Table 1). In addition, TV and its SDS and thyroid and hepatic haemodynamic parameters were significantly higher among ascitic than nonascitic group with a non significant difference regarding thyroid functions (Table 2). Moreover, all parameters did not significantly differ according to the etiology of liver disease (*P* > 0.05).

 On comparing all studied parameters in the 3 groups classified according to Child-Turcotte-Pugh classification [9], there was a progressive increase in the TV going from group A to C being highest in group C group. Also, there was a progressive decrease in fT_3_ going from group A to C (2.05 ± 0.2 in group A, 2.02 ± 0.2 in group B, and 1.8 ± 0.2 in group C, resp.). On the other hand, fT_4_ and TSH levels did not significantly differ between the 3 groups. Both ITAPI and ITARI increased progressively from group A to C (0.98 ± 0.1 in group A, 1.2 ± 0.11 in group B, and 1.9 ± 0.36 in group C resp. in ITAPI and 0.71 ± 7.1 in group A, 0.78 ± 3.0 in group B, and 0.88 ± 5.8 in group C, resp. in ITARI). Also, both HAPI and HARI increased progressively from group A to C (0.97 ± 6.2 in group A, 1.2 ± 0.11 in group B, and 2.0 ± 0.36 in group C, resp. in HAPI and 0.72 ± 5.3 in group A, 0.81 ± 4.7 in group B, and 0.89 ± 4.2 in group C, resp. in HARI). Thus, all thyroid and hepatic haemodynamic parameters increased with increased severity of liver disease. In addition, the frequency of increased TV, ITAPI and ITARI among cirrhotic patients increased progressively going from group A to C (Table 3).

**Figure 1:**
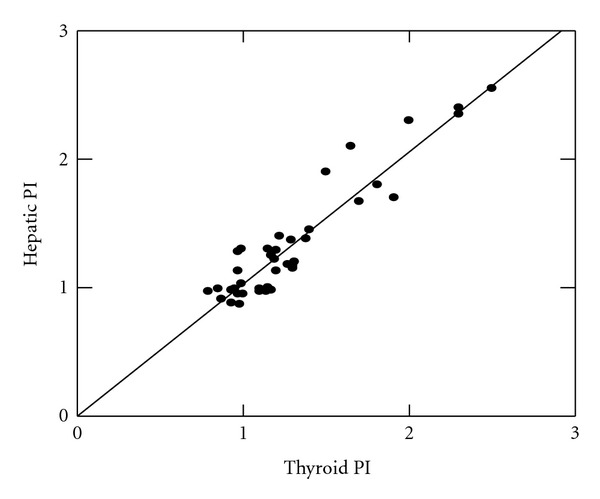
Correlation between hepatic and inferior thyroid arteries pulsatility indices among children with liver cirrhosis.

**Figure 2:**
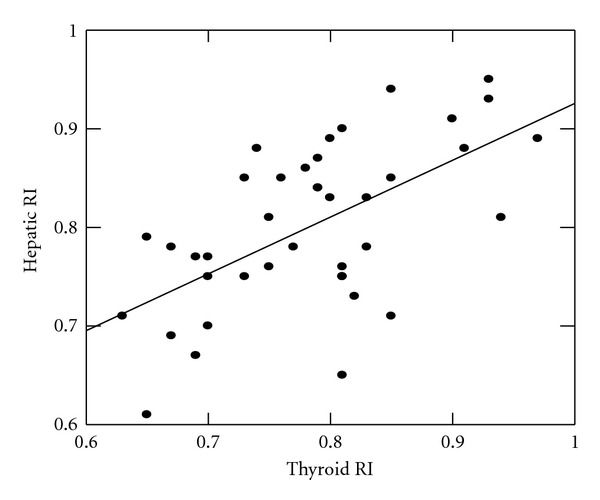
Correlation between hepatic and inferior thyroid arteries resistance indices among children with liver cirrhosis.

 Also, there were significant positive correlations between thyroid and hepatic arterial indices [ITAPI, and HAPI (*r* = +0.62, *P* = 0.03, Figure 1); ITARI and HARI (*r* = +0.42, *P* = 0.04, Figure 2)]. On the other hand, there was an inverse correlation between fT_3_ and each of ITAPI (*r* = −0.71, *P* = 0.021) and ITARI (*r* = −0.79, *P* = 0.011) while fT_4_ and TSH did not correlate significantly with each of ITAPI and ITARI (*P* > 0.05). Moreover, there were significant positive correlations between fT_3_ and each of serum albumin (*r* = 0.79, *P* = 0.02) and total proteins (*r* = 0.73, *P* = 0.025) and significant negative correlations between fT_3_ and each of ALT (*r* = −0.81, *P* = 0.01) and AST (*r* = −0.83, *P* = 0.01).

## 4. Discussion

Liver cirrhosis is characterized by complex changes of systemic and splanchnic haemodynamics. High-cardiac output and low-peripheral resistances are the features of the so-called hyperdynamic syndrome [2]. Portal hypertension is associated with hyperdynamic circulation with decreased arterial pressure, increased cardiac output, and splanchnic arteriolar vasodilatation causing blood congestion [4]. By contrast, in other areas such as the kidney and the thyroid gland, the resistance to arteriolar flow increases and blood perfusion of the organ decreases progressively [5].

 In the current study, TV was higher among patients and increased progressively as the severity of cirrhosis increased. Moreover, TV and its SDS were significantly higher in ascitic group. Our results agree with those of Hegedus, and his associates [16] who suggested that TV could be used in assessing severity of liver cirrhosis in addition to Child-Turcotte-Pugh classification. Factors responsible for increased TV in nonthyroidal diseases are not completely known, it increases with age and body weight, and some investigators demonstrated that lean body mass is a major determinant of thyroid size. Moreover, another study [17] suggested that modification in circulating thyroid hormone levels could be responsible for volume abnormalities in cirrhotic patients which is not the case in our study as TSH did not differ between cases and controls.

 In the current study, fT_3_ was significantly lower in patients than controls with a nonsignificant difference between ascitic and nonascitic groups which was in agreement with other studies [16, 17]. The liver has a primary influence on circulating levels of thyroid hormones. Most of the metabolically active thyroid hormone, T_3_, is generated in the liver from T_4_ through a selenium-dependent 5 deiodinase. Another selenium-independent deiodinase acts on the phenolic ring of T_4_ to produce the hormonally inactive reverse T_3_ (rT_3_) [18]. In cirrhosis, the most frequent abnormalities described involving thyroid function is the “low fT_3_ syndrome” with increased rT_3_ and decreased T_3_: T_4_ ratio [19]. Several mechanisms may be responsible for these abnormalities. In animal models, ethanol intake is associated with impaired hepatic 5 deiodination, suggesting that impaired hepatic deiodinase activity with decreased conversion of T_4_ to T_3_ and rT_3_ to T_2_ could take part in the modification in circulating hormone levels described in cirrhosis [20]. High serum rT_3_ levels have been demonstrated in cirrhotic patients with a high degree of liver damage [21]. Moreover, since the liver is the site of synthesis and degradation of carrier proteins thyroxin binding globulin (TBG), thyroxin-binding prealbumin (TBPA) and albumin, it is well-established that defective hepatocellular uptake and inefficient production of TBG are present in cirrhosis. Thyroid hormone concentration is also affected by glucagon levels and, in liver cirrhosis, plasma glucagon concentration is frequently elevated [22]. A significant negative correlation between plasma glucagon levels and serum T_3_, was found suggesting a role of hyperglucagonemia in the pathogenesis of low T_3_ values in these patients [23]. Thyroid dysfunction has been reported previously in a variety of nonthyroid illnesses including liver cirrhosis. Low total and free T_3_ with normal total T_4_ and TSH concentration in the absence of clinical hypothyroidism have been frequently reported in patients with nonthyroidal illnesses. Moreover, an inverse correlation between serum T_3_ concentration and the severity of liver dysfunction was reported [24], which was confirmed in the current study by the inverse correlation between fT_3_ and each of ALT and AST. Also, progressive decrease in T_3_ level in chronic liver disease has been described as indicative of a poor prognosis [25]. Moreover, poor nutritional factors is implicated in the low T_3_ syndrome in such patients as indicated by the positive correlation between fT_3_ and each of serum albumin and total proteins in our study. On the other hand, in 2001, Desi and his coworkers [26] found that there was an increase in serum-free T_3_ in patients with liver cirrhosis in relation to controls, which is against our results. This difference might be caused by the fact that the patients included in their study may have had an underlying thyroidal illness which was not excluded by the authors. Also, Gardner and his associates [27] found a nonsignificant difference in serum free T_3_ between cirrhotic patients and controls. This difference might be caused by the fact that the patients included in their study had less severe liver failure than those included in our study.

 In our series, fT_4_ and TSH did not differ between patients and controls which was also confirmed by other authors. [7, 16, 17, 27]. The latter findings suggest that the volume changes of the thyroid gland progressing with advanced stage liver cirrhosis reflect the metabolic changes of the “low fT3 syndrome” as a direct consequence of metabolic liver failure. On the other hand, Yamanaka and his associates [28] found an increase in free T_4_ level in patients with liver cirrhosis whose cause was acute hepatitis but there was a nonsignificant difference in serum-free T_4_ and TSH in patients with liver cirrhosis due to other causes when compared to controls. They explained this by the fact that increased T_4_ might be attributed to the viral infection that could cause thyroiditis and raised fT_4_.

Both ITAPI and ITARI were significantly higher in patients than controls with a progressive increase in the frequency of their rise going from group A to C, that is, with increased severity of liver disease. The reason for the increase in ITAPI and ITARI in cirrhotic patients is the vasoconstriction in thyroid circulation which is a part of the same haemodynamic picture of cirrhotic portal hypertension, secondary to increased vasoconstricting hormones. Also, the inverse correlation between fT_3_ levels and thyroid Doppler indices suggest that thyroid hormones changes could directly participate in haemodynamic alterations in cirrhotic patients suggesting their role in pathophysiology [7]. Thyroid hormones, particularly T_3_, decrease systemic vascular resistance by dilating resistance arterioles of the peripheral circulation through a direct effect on vascular smooth muscles. This vasodilating effect is attenuated by endothelial denudation, cyclooxygenase inhibition, and NOS (nitric oxide synthetase) inhibition that occur in cirrhosis. The latter hypothesis confirms the involvement of low fT_3_ in thyroidal arteries vasoconstriction in cirrhotic patients [29]. Moreover, there was a progressive increase in ITAPI and ITARI with a progressive increase in the frequency of their rise going from Child A to C groups, that is, with increased severity of liver disease. Also, ITAPI and ITARI were significantly higher in ascitic than nonascitic patients. Langer and his coworkers [20] found that ITAPI and ITARI increased in decompensated liver cirrhosis more than in compensated cases which goes with our results.

 Moreover, in our patients, Doppler indices in inferior thyroid artery correlated directly with hepatic arterial indices which was confirmed by other authors [16]. This suggests a role of portal hypertension in thyroid haemodynamic alterations since portal hypertension causes systemic vasodilatation which leads to activation of compensatory vasoconstricting mechanisms, in particular, the sympathetic nervous system, the rennin- angiotensin-aldosterone, and endothelin-1, and thus decreasing the vasodilating effect of thyroid hormones causing thyroidal arteries vasoconstriction [30].

 In addition, our series revealed higher HAPI and HARI in patients than controls. Schenk and his collegues [31] explained this by the fact that the hepatic arterial blood flow was lower and, thus, the hepatic vascular resistance was higher in cirrhotic patients than in controls which agrees with our finding. In addition, Cremona et al. 2001 [6] suggested that loss of functioning hepatic tissue, pathological changes which occur in liver cirrhosis such as distortion of the hepatic vascular bed by fibrosis, regeneration, collagenization of Disse space, and hepatocyte swelling may contribute to increased HAPI and HARI. In 1999, Shah and his coworkers [32] also suggested that in cirrhotic patients, there are multiple derangements in endothelial NOS-derived NO generation that contribute to impaired sinusoidal relaxation and increased intrahepatic resistance. Moreover, HAPI and HARI increased progressively as the degree of liver cirrhosis increased being higher in ascitic than nonascitic patients. Similarly, Schneider et al. [33] found a significant correlation between each of HAPI and HARI and Child-Pugh score, a prognostic parameter characterizing the degree of hepatic dysfunction in cirrhosis which also agrees with our findings and was confirmed by other authors. [7, 16, 23, 34] On the other hand, Vassiliades et al. 2004 [35], failed to prove a relationship between hepatic indices and the degree of liver cirrhosis while Alpern et al. [36] found the increase in HARI to be specific for cirrhotic patients with portal hypertension only.

 On the other hand, Doppler haemodynamic parameters and thyroid function tests did not significantly differ according to the etiology of liver disease meaning that the different pathogenetic mechanisms of liver cirrhosis have no influence on the hepatic/thyroid gland hemodynamic interaction.

In conclusion, the thyroid gland is involved, primarily and secondarily, in the haemodynamic alterations of cirrhosis; a reduction in vasodilator fT_3_ may play a role in the pathophysiology. Therefore, thyroid functions should be performed in patients with liver cirrhosis to detect the early changes in thyroid hormones and to offer proper treatment if needed. Also, study of the thyroid gland by Doppler could be useful in patients with liver cirrhosis to assess severity of liver cirrhosis in addition to Child-Turcotte-Pugh classification in order to predict its prognosis.

## Figures and Tables

**Table 1:** Comparison of Doppler haemodynamic parameters and thyroid function tests among cirrhotic patients and controls.

Variable	Patients (*n* = 40)	Controls (*n* = 30)	*t*/*z* ^#^	*P*
TV (mL)	7.9 ± 1.9 (3.3–13)	5.7 ± 0.39 (3.1–7.4)	2.6	0.042*
TV SDS	+2.8 ± 1.2 (+1.2 to +3.5)	+0.98 ± 0.48 (−0.5 to +1.63)	7.8^∗^	0.001**
ITAPI	1.2 ± 0.42 (0.8–2.5)	0.78 ± 5.7 (0.7–0.9)	4.6	0.001**
ITARI	0.78 ± 8.6 (0.63–0.77)	0.48 ± 2.1 (0.23–0.67)	7.1	0.041**
fT_3_ (pg/mL)	1.9 ± 0.2 (1.4–2.4)	3.9 ± 1.0 (2.7–6)	4.8	0.001**
fT_4_ (ng/dL)	1.6 ± 0.4 (0.8–2.3)	1.49 ± 0.5 (0.8–2.2)	0.7	0.4
TSH (mIU/mL)	4.05 ± 1.4 (1.7–7.5)	3.5 ± 1.4 (1.5–5.4)	1.04	0.3
HAPI	1.3 ± 0.46 (0.8–2.5)	0.74 ± 4.6 (0.68–0.81)	4.8	0.001**
HARI	0.8 ± 8.2 (0.6–0.9)	0.32 ± 5.4 (0.55–0.71)	6.2	0.001**

Results are expressed as mean ± SD or median and range, *z* test^#^, *P* < 0.05^∗^: significant, *P* < 0.01^∗∗^: highly significant, *P* > 0.05: non-significant, TV: thyroid volume, TV SDS: thyroid volume standard deviation score, ITAPI: inferior thyroid artery pulsatility index, ITARI: inferior thyroid artery resistance index, fT_3_: free triiodothyronine, fT_4_: free tetraiodothyronine, TSH: thyroid stimulating hormone, HAPI: hepatic artery pulsatility index, HARI: hepatic artery resistance index.

**Table 2:** Comparison of Doppler haemodynamic parameters and thyroid function tests among ascitic and non ascitic patients.

Variable	Ascitic (*n* = 12)	Non-ascitic (*n* = 28)	*t*/*z* ^#^	*P*
TV (mL)	10.9 ± 1.5 (8.9–13)	7.3 ± 1.6 (3.3–12)	3.1	0.020*
TV SDS	+2.6 ± 1.4 (+1.2 to +3.2)	+2.1 ± 1.3 (+1.0 to +3.1)	6.5^∗^	0.040*
ITAPI	2.16 ± 0.29 (1.8–2.5)	1.17 ± 0.25 (0.8–2)	3.4	0.032*
ITARI	0.87 ± 5.2 (0.80–0.93)	0.71 ± 8.1 (0.63–0.97)	2.8	0.048*
fT_3_ (pg/mL)	1.8 ± 0.2 (1.5–2)	2.0 ± 0.24 (1.4–2.4)	1.8	0.06
fT_4_ (ng/dL)	1.4 ± 0.49 (0.9–2.1)	1.6 ± 0.46 (0.8–2.3)	0.9	0.37
TSH (mIU/mL)	4.2 ± 1.4 (1.7–5.4)	4.0 ± 1.4 (1.7–7.5)	0.3	0.76
HAPI	2.16 ± 0.38 (1.7–2.55)	1.2 ± 0.33 (0.87–2.30)	3.3	0.029*
HARI	0.91 ± 2.5 (0.88–0.94)	0.78 ± 7.6 (0.61–0.85)	3.5	0.035*

Results are expressed as mean±SD or median and range, *z* test^#^, *P* < 0.05^∗^: significant, *P* > 0.05: nonsignificant, TV: thyroid volume, TV SDS: thyroid volume standard deviation score, ITAPI: inferior thyroid artery pulsatility index, ITARI: inferior thyroid artery resistance index, fT_3_: free triiodothyronine, fT_4_: free tetraiodothyronine, TSH: thyroid stimulating hormone, HAPI: hepatic artery pulsatility index, HARI: hepatic artery resistance index.

**Table 3:** Frequency of thyroid Doppler haemodynamic abnormalities among cirrhotic patients.

Variable	Group A (*n* = 15)	Group B (*n* = 15)	Group C (*n* = 10)
Increased TV	3 (20%)	12 (80%)	9 (90%)
Increased ITAPI	4 (26.6%)	13 (86.6%)	10 (100%)
Increased ITARI	5 (33.3%)	15 (100%)	10 (100%)

Results are expressed as frequency and percentage. TV: thyroid volume, ITAPI: inferior thyroid artery pulsatility index, ITARI: inferior thyroid artery resistance index.
